# The Role of Protein-Rich Extracts from *Chondrus crispus* as Biostimulant and in Enhancing Tolerance to Drought Stress in Tomato Plants

**DOI:** 10.3390/plants12040845

**Published:** 2023-02-13

**Authors:** Guido Domingo, Milena Marsoni, Milena Álvarez-Viñas, M. Dolores Torres, Herminia Domínguez, Candida Vannini

**Affiliations:** 1 Biotechnology and Life Science Department, University of Insubria, 21100 Varese, Italy; 2 CINBIO, Facultade de Ciencias, Universidade de Vigo, Campus Ourense, 32004 Ourense, Spain

**Keywords:** red seaweed, green extraction, biostimulant, drought, hydrolysed protein

## Abstract

The application of seaweed extract-based biostimulants is a promising approach for achieving sustainable agriculture, with an enormous potential of improving crop yield and mitigating climate change effects. Abiotic stressors, such as drought, are major factors resulting in tomato (*Solanum lycopersicum* L.) yield losses and seaweed-based biostimulants have been proposed as an eco-friendly strategy to counteract this negative impact. *Chondrus crispus* is a common red seaweed widely used as source of carrageenans, not yet explored as a plant biostimulant. In this study, a protein hydrolysate-rich *C. crispus* extract, by-products of the carrageenan extraction, was tested on tomato plants under well-watered condition and water shortage. The foliar application of the protein-rich *C. crispus* extract conferred drought tolerance to tomato plants resulting in less noticeable visual stress symptoms. Treated plants showed higher shoot height and biomass under both well-watered and water deficit conditions, evidencing the double effect exerted by this new biostimulant, as plant growth promoter and drought stress protector. The treatment with the biostimulant had an effect on levels of abscisic acid and proline, and triggered the expression of Solyc02g084840, a drought marker gene. Finally, a label-free mass spectrometric approach allowed us to identify phycoerythrins and phycocyanins as major bioactive proteins contained in the extract. Altogether, these results indicate that the foliar application of protein hydrolysate-rich extracts from *C. crispus* improved tomato plant growth and tolerance to drought stress, suggesting a new opportunity for further applications in the agriculture and horticultural sectors.

## 1. Introduction

Recently, the agricultural sector is facing multiple simultaneous challenges, by rising the productivity to feed the growing global demand and coping with the climate change which is disrupting weather patterns [1]. Hotter temperatures and changing rainfall patterns lead to an altered water supply, thus generating plant drought stress which has recently become a major constraint to agriculture [2]. Unfortunately, part of the conventional agriculture still relies on agrochemicals (e.g., synthetic fertilizers and pesticides) but the use of more sustainable methods in the agriculture production systems is needed [3].

To date, the application of biostimulants in agriculture has shown huge potential not only to improve crop yield and quality, reducing fertilizer requirements, but also to enhance plants resistance to stresses related to climate change, such as drought [4,5,6]. In this regard, seaweeds are amazing sources of bioactive compounds, useful to promote plant growth, increase yields and plant’s tolerance to abiotic and biotic stresses. A number of seaweeds are reported to possess plant-growth promoting activity [7]. Undoubtedly, *Ascophyllum nodosum* is one of the most widely researched seaweed in this area. *A. nodosum* extracts have been demonstrated to increase plant growth, also in the presence of abiotic and biotic stresses [8].

Despite this great interest, there is still debate about which compounds present in the seaweed extracts are involved in the positive effects induced. In fact, seaweed extracts are a heterogeneous group of compounds including minerals, polysaccharides, polyunsaturated fatty acids, bioactive peptides and proteins, amino acids, and phytohormones [7,9,10] which could act on plant metabolism in a specific or non-specific manner [11,12]. Among major bioactive components present in seaweed extracts, polysaccharides such as carrageenans, alginates and their oligo-derivatives are well-known as plant biostimulants and elicitors of defense responses [13,14,15]. Trivedi et al. [16] identified glycine betaine, choline chloride, and zeatin as the major plant biostimulants in *Kappaphycus alvarezii* seaweed extract, with a protective role in maize. Protein hydrolysates, containing peptides and amino acids, are another class of plant biostimulants that can alleviate the negative effects due to salinity, drought and heavy metals stresses [17]. Plant-derived protein hydrolysates are attractive as plant biostimulants due to their potential to increase the germination, productivity and quality of a variety of crops [18]. In a recent study performed on sweet basil, Rouphael et al. [19] confirmed that the vegetal protein hydrolysates offered better results over the animal derived ones, increasing photosynthesis and color status, ions content, yield, and quality.

Different methods, both chemical (solvents, acids, and alkali) and physical (heat, pressure, and microwaves), have been proposed to obtain bioactive compounds from seaweed extracts [7]. Protein hydrolysates from seaweed-derived raw materials are usually produced using enzymatic and/or chemical methods [17]. Among the most environmentally sustainable processes, the microwave assisted heating was successfully used for the rapid water extraction of carrageenans and other bioactive compounds from *Mastocarpus stellatus* [20]. In a previous work [21], we used a hydrothermal treatment with subcritical water as green extraction technology for the extraction of carrageenans from *Chondrus crispus*.

*C. crispus*, commonly known as ‘Irish moss’, is an edible red seaweed widely used as source of carrageenans, not yet explored regarding its biostimulant properties. The use of pressurized hot water extraction under subcritical conditions proved to be an efficient, chemical free, tunable and rapid process to solubilize carbohydrates, protein and phenolics. In fact, after the extraction process, carrageenans were recovered generating a soluble by-product that still contained bioactive compounds. In particular, the use of temperatures between 140 and 160 °C has proven to be effective to obtain fractions rich in hydrolyzed proteins of high biological value.

In this study, by using a label-free LC-MS/MS analysis, we characterized the hydrolyzed protein extracts obtained by the hydrothermal treatment of *Chondrus crispus* with subcritical water at different temperatures (120–200 °C). The biostimulant properties of the *C. crispus* extract richer in proteins was subsequently tested on tomato (*Solanum lycopersicum* L.), one of the most widely cultivated and economically important crop plants worldwide, very sensitive to water deficit [22].

## 2. Results

### 2.1. Proteins Contained in C. crispus Extracts Are Mainly Pigment-Protein Complexes

The determination of total protein concentration by the Bradford method revealed that the free-carrageenans extracts obtained at 120 and 160 degrees were the richest in protein content (26.52 and 25.68 g/100 g, respectively; Table S1). Our analysis confirmed previous findings [21] revealing that in the extracts obtained at 160 °C the number of proteins identified was the higher. In fact, after the label-free LC-MS/MS analysis and data processing, a total of 58, 68, 170, 26, and 4 proteins were consistently identified in at least two biological replicates of *C. crispus* extracts obtained at 120, 140, 160, 180 and 200 °C, respectively (Table S2).

Among the most abundant proteins identified, the majority was pigment-protein complexes, such as phycoerythrins and phycocyanins. These aggregates of light-harvesting proteins constitute the phycobilisomes, the light-harvesting antennae in cyanobacteria and red algae [23]. All identified proteins, grouped by functional categories, resulted mainly involved in photosynthesis, protein folding and ATP production.

The top 10 proteins by label free quantification (LFQ) identified in extract obtained at 160 °C were listed in Table 1. The mean length of peptides detected in this sample was 12.1 amino acid residues, while their mean molecular mass was 1311 Da (Table S3).

### 2.2. C. crispus Extract Treatment Stimulates Growth of Tomato Plants in Control Conditions and under Water Deficit

**Figure 1:**
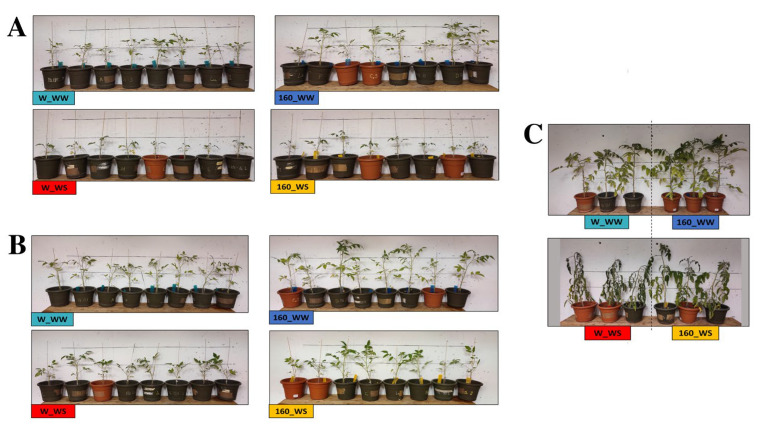
Phenotypic effects of the *C. crispus* extract foliar application on tomato plants grown in the absence (W_WW, 160_WW) and presence of drought stress (W_WS, 160_WS): (**A**) after the first water deficit (T1); (**B**) after the two day of recovery (T2); (**C**) after the second water deficit (T3). Well-watered untreated plants (W_WW); well-watered plants treated with the *C. crispus*-extract (160_WW); water-stressed untreated plants (W_WS); water-stressed plants treated with the *C. crispus*-extract (160_WS).

**Figure 2:**
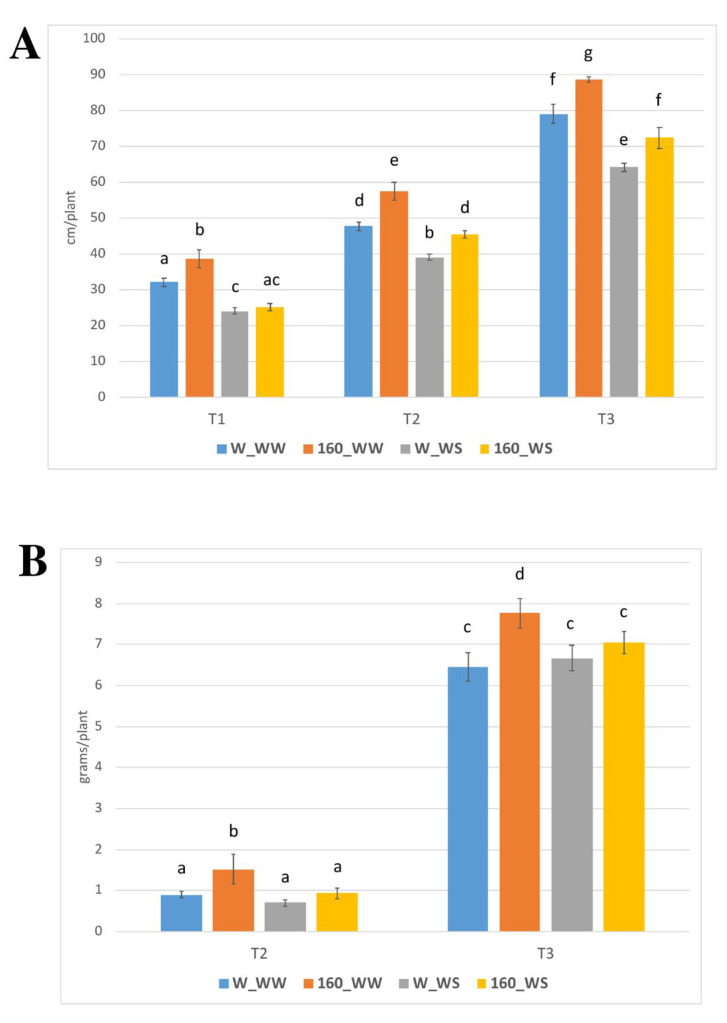
Effect of protein-rich extracts from *C. crispus* on the shoot height (**A**) and the dry weight ((**B**); DW) of tomato plants foliar sprayed two times and subjected to two water stress treatments (T1, T3) separated by a recovery period (T2). Data are means ± SE. Different lowercase letters indicate significant differences at the *p* < 0.05 level obtained by using two-way ANOVA and Tukey’s HSD test.

The foliar application of *C. crispus* extract (160-degree extract; 2 g/L + 0.1% Tween) promoted shoot growth in the control as well as in the stress condition (Figure 1 and Figure 2). Compared to unstressed control, *C. crispus* extract increased 20, 20 and 12% shoot under well-watered condition at three time points considered, respectively (Figure 2A). Under water stress condition and still compared to stressed control, 4 and 16% increases were observed after the first water stress (T1) and after the two days recovery (T2), respectively. *C. crispus* extract also led to a significant higher plant height of 13% after the second water stress (T3).

Shoots were collected after recovery (T2) and second drought treatment (T3), then biomasses were determined (Figure 2B). Under control condition and compared to unstressed control, 70 and 20% statistically significative increases were observed both at T2 and T3, respectively.

After the second water stress (T3; Figure 1C), a severe wilting of all the leaves was reported in control plants (W_WS), while *C. crispus*-treated plants showed less noticeable visual stress symptoms (160_WS).

### 2.3. C. crispus Extract Increases Endogenous ABA Levels

**Figure 3:**
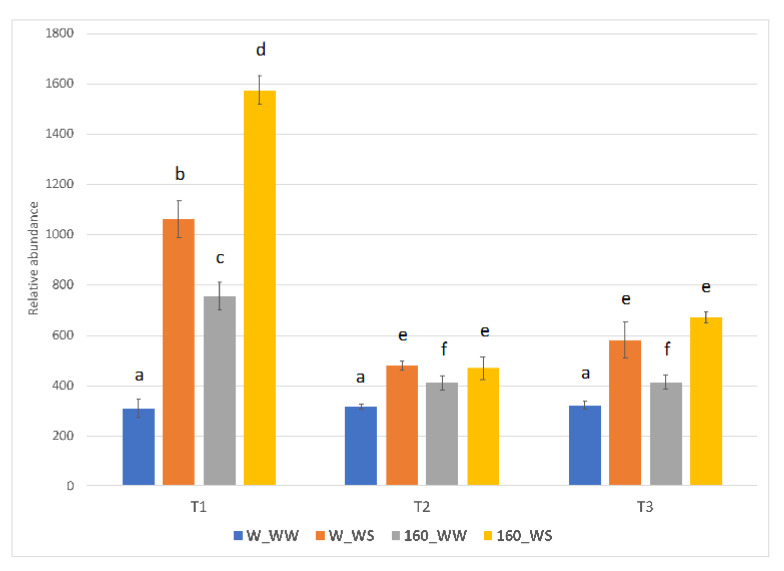
Effect of the protein-rich extract from *C. crispus* on ABA content in tomato plants following first water deficit (T1), two day of recovery (T2) and a second water deficit period (T3). Data are means ± SE expressed as relative intensities among samples. Different lowercase letters indicate significant differences at the *p* < 0.05 level obtained by using two-way ANOVA and Tukey’s HSD test.

To determine whether drought tolerance of *C. crispus*-treated tomato was associated with a change in the abscisic acid (ABA) levels, ABA content was measured at three time points considered (T1–T3; Figure 3). In untreated plants, ABA level was increased by 3.44, 1.51 and 1.81-fold at T1, T2 and T3, respectively, as compared to unstressed condition. Interestingly, *C. crispus* treatment increased the ABA level already in control condition (2.46, 1.31 and 1.81-fold as compared to untreated condition at T1, T2 and T3, respectively), and further increased ABA concentration after the first water deficit stress (5.11-fold with respect to W_WW plants at T1).

### 2.4. C. crispus Extract Treatment Modulates Proline Levels in Tomato Plants

**Figure 4:**
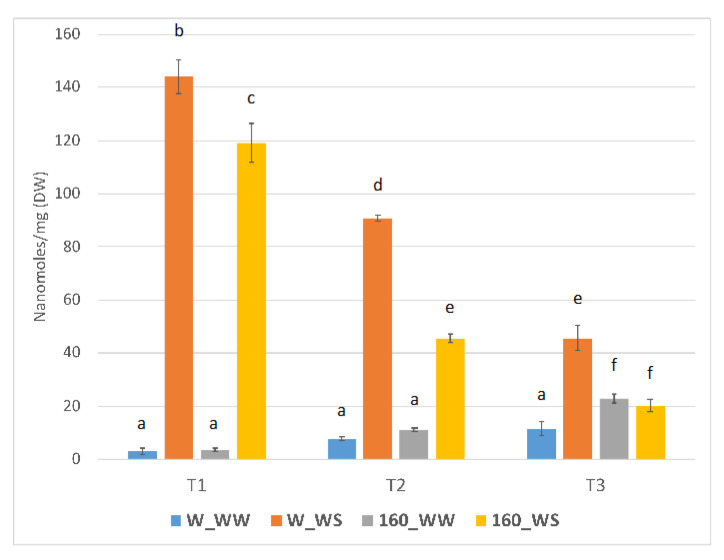
Effect of the protein-rich extract from *C. crispus* on free-proline content in tomato plants following first water deficit (T1), two day of recovery (T2) and the second water deficit period (T3). Data are means ± SE expressed as relative intensities among samples. Different lowercase letters indicate significant differences at the *p* < 0.05 level obtained by using two-way ANOVA and Tukey’s HSD test.

The accumulation of an osmo-protectant similar to proline was evaluated at the three sampling points considered (T1, T2, T3; Figure 4). After drought stress, proline levels of untreated plants were higher with respect to unstressed conditions, at all time points considered. In these plants, the first water deficit stress induced a 46.5-fold higher proline accumulation that reduces to 11.4-fold after two days of recovery. The second water stress led to a 3.95-fold higher proline accumulation. *C. crispus*-treated plants had 32.6 and 4.04-fold higher proline accumulation at T1 and T2, respectively, as compared to the unstressed condition (160_WW). A proline accumulation trend is also observed in *C. crispus*-treated but unstressed plants which becomes significant at T3 (1.98-fold increase with respect to W_WW). The second water stress did not lead to a significant increase in the level of proline in the treated plants.

### 2.5. C. crispus Extract Treatment Modulates the Expression of a Stress Protective Gene under Drought

**Figure 5:**
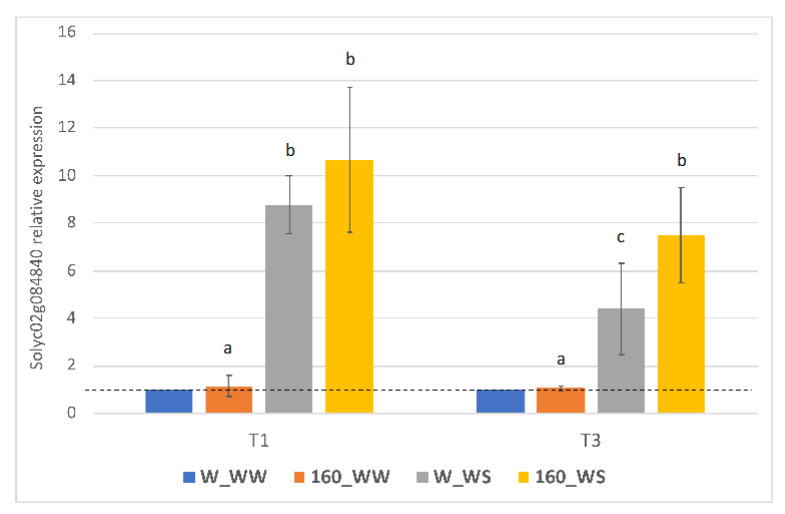
Gene expression analysis of Solyc02g084840 gene after first (T1) and second (T2) water deficit periods. Relative expression of Solyc02g084840 gene was reported as the number of fold increase of the transcript level at each time point, compared to the control condition (W_WW; dotted line). Three biological samples per treatment were analyzed with three technical replicates per sample. Data are means ± SE expressed as relative intensities among samples. Different lowercase letters indicate significant differences at the *p* < 0.05 level obtained by using two-way ANOVA and Tukey’s HSD test.

In order to examine whether drought stress and *C. crispus* extract affected the regulation of stress responsive genes at the transcriptional level, relative changes in Solyc02g084840 gene expression were analyzed by qRT-PCR (Figure 5). Solyc02g084840 transcript was strongly induced in leaves of treated and untreated plants (up to more than 8-fold) after first drought stress (T1). A positive regulation was also observed after the second water deficit (T3): untreated plants showed a significative lower induction (4.39-fold) than those treated with the *C. crispus* extract (7.49-fold).

## 3. Discussion

To reduce the problems associated with synthetic agrochemicals, attention has recently turned to both foliar and soil application of natural biostimulants which has been proved to improve physiology and metabolism of crops, and consequently their growth [24]. Biostimulants are also an emerging class of crop management products useful to enhance crop resistance to environmental stressors. In this regards, plant-derived protein hydrolysates represent an innovative technology with promising application potential [17] and seaweed extracts represent one of the fastest growing biostimulant category [25,26,27]. To date, many seaweed-based extracts are known for their biostimulant properties. However, to the best of our knowledge, no studies report the application of extracts from *Chondrus crispus*. Our results, here for the first time, supported the biostimulant and bioprotective effect exerted by the foliar application of a protein-rich *C. crispus* extract, by-product of the carrageenan extraction.

According to Alvarez-Vinas and colleagues [21], *C. crispus* extracts generated using temperatures of 140 and 160 °C were richest in hydrolyzed proteins. Our gel- and label-free confirmed those results by identifying the largest number of proteins in the 160-degree extract. This protein-rich extract was foliar sprayed twice on tomato plants exposed to two consecutive water deficit conditions, in order to unravel its biostimulant properties.

When treated with a foliar application of *C. crispus* extract (2 g/L), a significant difference in tomato plants growth and DW was recorded, as compared to untreated conditions. Under well-watered conditions, the increase was always greater than 12%, at the three time points considered, while under water deficit increases equal to 4%, 16 and 13% were observed after the first water stress, after the two days recovery, and after the second water stress, respectively. A similar trend was observed for DW but with statistically significant increases due to the *C. crispus* extract applications only under control condition. Phenotypic observations confirmed the growth promotion achieved in treated tomato plants, under both stressed and non-stressed conditions and compared with the untreated plants. It is generally accepted that one of the first visual symptoms of drought in plants is the leaf wilting. The foliar application of the *C. crispus* extract prevents this phenomenon which is clearly visible in the stressed but not treated plants (W_WS; Figure 1C).

To further investigate the beneficial effects of biostimulants in plant tolerance to drought, ABA and proline content were quantified. Proline is usually increased under stress conditions playing osmoprotectant and antioxidant roles, mainly in drought and salinity stress [28]. In the present study, an expected increase in free proline content was observed in leaf tissues of untreated plants subjected to water deficit, with a decidedly greater increase following the first drought stress. Rising levels of proline were also recorded in plants treated with *C. crispus* extracts but only after the first water deficit. However, a proline accumulation trend was also observed in unstressed plants treated with *C. crispus* extract with respect to control plants. Following the second treatment, treated plants had significantly higher levels of endogenous proline than the control. This could explain, at least in part, the better resistance of treated plants to the second water deficit, as evident from the minor symptoms reported (Figure 1C).

The lower free proline increase recorded after the first stress (T1) in treated tomato plants may be an indicator of a lower stress perceived. Moreover, several reports indicate that the effects of proline depend on its levels and the concentrations necessary to exert a beneficial effect are relatively low. In fact, when applied exogenously, proline at a low concentration (e.g., 30 mM) has positive effect increasing stress tolerance, whereas at higher concentrations (e.g., 40–50 mM) proline resulted in toxic effects and poor plant growth [29,30].

Our data indicate that the foliar application of *C. crispus* extract was also able to regulate abscisic acid level in tomato plants. Already in control condition, the *C. crispus* treatment increased the ABA level as compared to the untreated conditions. Moreover, a higher increased in ABA concentration was observed after the first water deficit stress with respect to stressed but untreated tomato plants.

A better performance under water deficit conditions was recorded in plants with higher ABA levels [31,32,33]. In fact, the activation of the ABA signaling rapidly closes the leaf stomata preventing water loss [34] and reprograms gene expression to alleviate drought effects [35]. The application of seaweed extracts to crops increases the ABA level, together with the expression of abscisic acid-responsive genes [33,36,37]. The foliar application of an alginate oligosaccharide induced the expression of ABA-responsive genes linked to drought resistance [38]. Although ABA or ABA-like compounds have been detected in some seaweed extracts, their concentration is believed too low to elicit a physiological response [39].

To support these results and deeply investigate the molecular mechanisms underlying the biostimulant and bioprotective effects exerted by the *C. crispus* extract, the transcript level of Solyc02g084840, a drought marker gene encoding for a dehydrin and triggered by osmotic stress [40], was investigated. Dehydrins, also known as group II late embryogenesis abundant (LEA) proteins, are multi-family proteins usually induced under cold and drought stress and associated to important protective functions in plants [41,42]. *RAB18* (Arabidopsis ortholog of the Solyc02g084840 gene) encodes for a glycine-rich hydrophilic protein [43] that prevents protein denaturation [44] and protects membranes under dehydration conditions by binding to anionic phospholipids [45]. Under drought and salinity stress seaweed biostimulants based on *A. nodosum* are described to modulate the expression of several dehydrins genes, including *RAB18* [8,36]. Moreover, when *RAB18* is downregulated, Arabidopsis and rice plants displayed poor drought tolerance [46].

In our study, the Solyc02g084840 transcript increased rapidly after the two drought stress periods, in both treated and untreated plants. However, following the second water deficit, the induction observed was higher in tomato plants treated with the *C. crispus* extract with respect to the untreated plants. These data support the mitigating effect induced by the foliar application of *C. crispus* extract in tomato plants subjected to a second water deficit.

The seaweed-based biostimulants are not a homogenous category; their composition varies depending on the seaweed species used and/or the extraction process [47,48]. Their biochemical composition includes both molecules of small dimension such as hormones, antioxidants and pigments and more complex molecules such as polysaccharides, peptides and proteins [47,49,50].

The characterization of carrageenan-free extracts from *C. crispus* used in this study (160-degrees extracts) showed high concentration of hydrolyzed proteins and lower levels of sugars, phenolics and minerals [21]. Several studies reported the positive effects induced by the applications (both soil and foliar) of plant-derived protein hydrolysates on growth, yield and fruit quality of crops [17,51,52,53,54,55]. However, regarding tomato cultivation, limited information is still available. Nevertheless, the foliar application of a legume-derived protein hydrolysates increased growth, yield and fruit quality in two greenhouse tomato cultivars [56]. Soy protein hydrolysates improved tomato plant fitness, fruit yield, and resistance to biotic stress [57]. However, the complete characterization of peptides and proteins present in plant-based hydrolysates used as biostimulants is often missing.

Soluble peptides and free amino acids, derived from the hydrolysis of proteins, could act as signaling molecules [17]. Peptides present in plant-based protein hydrolysate may directly possess phytohormone-like activities by eliciting an auxin and gibberellin-like activity [55,58,59].

Evidences suggest that the small size of the peptides is important for their absorption and for their biostimulating effect [60,61]. Free amino acids and low-molecular size peptides can be easily absorbed by leaves and induce endogenous phytohormonal biosynthesis [60]. In the *C. crispus* extract obtained by the hydrothermal treatment at 160 °C we mainly identified low molecular weight peptides (average molecular weight of 1311 Da and 12 amino acid residues), supporting these data. Moreover, the label-free LC-MS/MS analysis allowed us to fully characterize the hydrolyzed proteins present in the *C. crispus* extract and thus identify some putative candidates involved in beneficial effects exerted. We detected large quantities phycoerythrins and phycocyanins, pigment-protein complexes that constitute the phycobilisomes and play a role in the harvesting of the light. To the best of our knowledge, few evidences related to the biostimulant properties of seaweed pigment-protein complexes have been reported. Phycocyanin-rich Spirulina extract (PRSE) application showed an increased yield by 12.5% and improved antioxidant flavonoid levels of lettuce grown hydroponically [62]. PRSE-treated lettuces were more vigorous reaching maturity 6 days (21% more rapidly than the untreated group). Moreover, the 5-aminolevulinic acid (ALA), the precursor of phycocyanins biosynthesis, has proven useful to increase plant growth and yield [63]. The exogenous application of ALA to kidney bean, barley, potato and garlic significantly increased their growth and yield by 10–60% [63,64]. The ALA growth-promoting effect was attributed to an improvement the photosynthetic activity in grapevines [65].

## 4. Materials and Methods

### 4.1. Extract Preparation and Characterization

#### 4.1.1. Hydrothermal Extraction with Subcritical Water

The hydrolyzed protein-rich extracts were obtained from *Chondrus crispus* (9.6 ± 0.3 g/100 g of moisture content; Compañía Española de Algas Marinas S.A., CEAMSA, Pontevedra, Spain) according to Alvarez-Viñas et al. 2022 [21]. Briefly, seaweeds were combined with distilled water at a ratio of 30:1 (*w*/*w*), placed in a pressurized Parr Instruments reactor (series 4842, Moline, IL, USA) and heated during 10–60 min until the selected temperature in the range of 120–200 °C was reached (i.e., 35 min were required in order to attain 160 °C). Next, the reactor was cooled down to room temperature and the suspension was filtered. Crude carrageenan was separated from the extract by ethanol precipitation. The determination of protein concentration in each *Chondrus crispus* extract was carried out by the Bradford method [66].

#### 4.1.2. LC-MS/MS Analysis

The proteins present in the carrageenan-free extracts were characterized by LC-MS/MS analysis. Protein samples were loaded onto a standard Laemmli-type polyacrylamide gel, allowed to stack and enter the resolving gel but not to separate. At the end of the run, gel pieces were excised and subjected to in-gel digestion. Briefly, gel pieces were washed sequentially with ammonium bicarbonate 25 mM and 50% ACN/ammonium bicarbonate 25 mM in an ultrasonic bath. Proteins were then reduced with 10 mM DTT for 1 h, and alkylated with 55 mM IAA for 30 min. The digestion was performed by using 40 ng of trypsin at 37 °C overnight. Finally, tryptic peptides were extracted twice from the gel matrix with 0.5% TFA and 100% ACN. Peptides were dried in a speed-vacuum concentrator at 45 °C (Concentrator plus, Eppendorf, Hamburg, Germany), reconstituted in LC/MS-grade water containing 0.1% (*v*/*v*) formic acid and analyzed by a hybrid high-resolution LTQ-Orbitrap Elite mass spectrometer coupled to a Proxeon Easy-nLC 1000 UHPLC system (Thermo Fisher Scientific, Waltham, MA, USA).

The peptides were loaded onto a reverse phase column (PepMap^®^ RSLC C18, 2 µm, 100 Å, 75 µm × 50 cm, Thermo Fisher Scientific, Waltham, MA, USA) and eluted with an ACN gradient of 5–30% containing 0.1% formic acid for 240 min. The tandem mass spectrometer scan was performed in positive ion mode, with top 15 at 35% normalized collision energy and a dynamic exclusion time at 30 s. The minimum signal threshold was set at 1000, the resolution at 30,000, and the isolation width at 2.0 Da. A full MS scan was performed from 390−1700 m/z with resolution at 120,000.

#### 4.1.3. Elaboration of Raw Data and Downstream Bioinformatic Analysis

Raw data were searched by using the MaxQuant program (v.1.5.3.3, http://www.coxdocs.org/doku.php?id=maxquant:start, accessed on 18 January 2023) against the *Chondrus crispus* database (downloaded on 1 June 2022 from www.uniprot.org). The search criteria were set as follows: two missed cleavages, fixed modification of cysteine (carbamidomethylation), variable modifications of methionine (oxidation) and phosphorylation on serine, threonine and tyrosine, minimum peptide length of six amino acids, precursor mass tolerance 4.5 ppm for the main search. The match between runs (time window of 0.7 min) and target-decoy search strategy (revert mode) options were enabled. A false discovery rate (FDR) of 1% was accepted for peptide and protein identification, respectively. The raw data obtained were processed using an in-house tool [67]. Incorrect identifications, such as contaminants and not consistent identifications, were filtered out. Only protein detected in least two of the three biological replicates in almost one analytical group were considered.

### 4.2. Plant Material and Growth Conditions

Tomato (*Solanum lycopersicum* cv. San Marzano Nano) seeds were obtained from local seed supplier. Seeds were germinated in the growth chamber (16 h light/8 h dark; 200 µm m^−2^ S^−2^). One week after germination (after the first two true leaves appearance) each plant was transplanted into 20 cm pots with a homogenous and equal weight of potting soil (Vigorplant Completo^©^; 23% coir pith, 27% Irish peat, slow-release fertilizer, 14% volcanic pumice, 36% superfine peat). Tomato plants were raised in a complete randomized design in a growth room at a temperature of 27/22 ± 2 °C (day/night; 16/8 h) and 70 ± 5% relative humidity (RH) under a light intensity of 120 μmol m^−2^ s^−1^. Plants were irrigated with 200 mL water every other day in order to create equal soil moisture conditions in all the pots.

### 4.3. Biostimulant Application and Drought Stress Conditions

**Figure 6:**
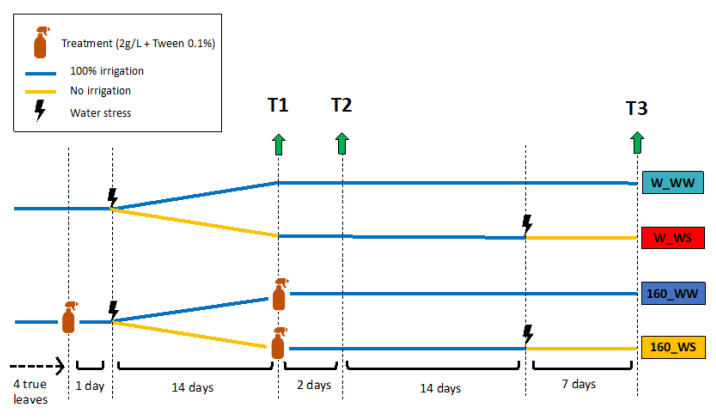
Schematic representation of the experimental design with the sampling time points for molecular and physiological analysis. Tomato plants were grown in the absence (W_WW, 160_WW) and presence of drought stress (W_WS, 160_WS) and sampled after first water stress period (T1), two day after recovery (T2) and at the end of the second water stress (T3).

A schematic representation of the experimental design was represented in Figure 6. The plants (N = 64) were randomly divided into two sets (set n.1, 32 plants and set n.2, 32 plants) and assigned to four different experimental groups: mock plants well-watered (W_ WW), mock plants with water deficit (W_WS), *C. crispus* extract-treated plants well-watered (160_WW), and *C. crispus* extract-treated plants with water deficit (160_WS) (Figure 6).

For the experiments, plants were foliar-sprayed twice with 10 mL of deionized water with 0.1% Tween (W_WW and W-WS plants) or 10 mL of a solution containing 2 g/L of freeze dried carrageenans-free *C. crispus* extract obtained at 160 °C with 0.1% Tween (160_WW and 160_WS): at the fourth true leaf appearance and 14 days after the first water stress. After the first biostimulant treatment, water stress was induced in half of the plants by stopping the water intake (W_WS and 160_WS). The other half continued to be well-irrigated (untreated plants; W_WW and 160_WW). The second biostimulant application was carried out at the end of the first stress. To minimize the influence of any positional effect on drought stress responses, the relative position of the pots in the growth room was changed every other day. Plant materials were collected after first stress period (T1; set n.1), two days after recovery (T2; set n.1) and at the end of the second water stress (T3; set n.2) (Figure 6). For each experimental group, samples were pooled and grinded into powder with a mortar and pestle with liquid nitrogen; 50 mg of homogenized samples were transferred to 2 mL screwcap and used for RNA extraction. The remaining powder was freeze dried lyophilized and stored at room temperature for further analyses.

### 4.4. Shoot Length and Biomass Measurements

Morphological changes in plants were observed by measuring shoot length at 14 days after the first stress (T1), two days after recovery (T2) and 7 days after the second stress (T3). The shoot part was cut at T2 (set n.1) and T3 (set. n.2) and the dry matter content was measured drying the material at 75 °C for 48 h to obtain the dry weight (DW).

### 4.5. Determination of ABA Content

The abscisic acid (ABA) content was determined as described by Mancini and colleagues [68], with three biological repetitions. Briefly, 20 mg of freeze-dried leaf material was ground into powder in a mortar by using liquid nitrogen. The ABA extraction was performed by adding 800 μL of 2-propanol/H_2_O/concentrated HCl (2:1:0.002, *v*/*v*/*v*). An Acclaim RSLC 120 C8 column (Thermo Scientific; 2.2 µm, 120 Å, 2.1 mm × 100 mm) and a gradient elution with a flow rate of 0.2 mL min^−1^ at 25 °C were used for the peptide separation. The mobile phase was composed of water with 0.1% (*v*/*v*) formic acid (solvent A), and methanol plus 0.1% (*v*/*v*) formic acid (solvent B). The gradient elution program was: 0–3.0 min/5% (*v*/*v*) B, 3–20 min/5–50% (*v*/*v*) B. The analyses were performed by using a Finnigan LXQ linear ion trap mass spectrometer equipped with an ESI ion source (Thermo Electron Corporation, Carlsbad, CA, USA) in the negative ion mode (spray voltage 2.5 kV, capillary temperature 250 °C) and in multiple-reaction monitoring (MRM) mode. MRM acquisition was accomplished monitoring the 263/153 transition. ABA contents were expressed as relative intensities among samples.

### 4.6. Determination of Proline Content

In this case, 30 mg of freeze-dried leaf material from three sampling times (T1, T2 and T3) and three biological repetitions were homogenized in 52 mL of ethanol 80% (*v*/*v*). Samples were shaken (100 r.p.m.) at 4 °C for 30 min and then boiled at 95 °C for 20 min. The homogenates were centrifuged at 20,000× *g* for 5 min at 4 °C. A total of 50 μL of supernatant was mixed with 100 μL of reaction mixture [1% (*w*/*v*) ninhydrin in acetic acid/water/ethanol (60/20/20, *v*/*v*/*v*)] and incubated for 20 min at 95 °C. After cooling at room temperature, the absorbance was measured at 520 nm in a microtiter plate using the Infinite 200 PRO instrument (Tecan, Life sciences, Männedorf, Switzerland). After preparing a calibration standard curve with L-proline (Sigma-Aldrich, St. Louis, MO, USA), proline content was expressed as nanomoles mg^−1^ DW.

### 4.7. RNA Extraction and Relative Gene Expression by qRT-PCR of Drought Stress Marker Genes

Total RNA was isolated from about 100 mg of frozen ground leaf material from three sampling times (T1, T2 and T3) by using TriFast™ (Euroclone, Pero, Italy) following the manufacturer’s instructions. RNA was treated with AMPD1 DNase I Kit (Sigma-Aldrich) in order to remove efficiently genomic DNA contamination. The RNA concentration and purity were measured in a Nanodrop™ spectrophotometer (Fisher Scientific) loading 1 µL of extracted RNA. The ratios of absorbance at 260 nm and 280 nm, and at 260 and 230 nm were used to assess the purity of RNA. The reading of the blank was carried out with 1 µL of RNase free water. RNA integrity was checked on a 1% agarose gel by checking the presence and the quality of 28S and 18S ribosomal RNA (rRNA) bands [69]. One microgram of the extracted RNA was used as template for the synthesis of cDNA, following the protocol of the iScript™ cDNA synthesis kit (Bio-Rad Laboratories, Hercules, CA, USA). The expression analysis of a tomato drought stress marker gene (Solyc02g084840) strongly upregulated by drought [70] was studied in the shoot tissue at T1, T2 and T3. Solyc02g084840 gene was also selected because orthologous of RAB18 gene, according to the subfamily information retrieved from the PANTHER 17.0 classification system (data retrieved on 1 October 2020 from http://www.pantherdb.org/panther/family.do?clsAccession=PTHR33346:SF42). The SlEF1A (*S. lycopersicum* elongation factor 1-alpha) gene was used as reference gene [71].

The efficiencies of target and housekeeping genes were determined by qRT-PCR on serial dilutions of RNA template over a 100-fold range [71], with similar results. Primers were designed by using primer-BLAST (https://www.ncbi.nlm.nih.gov/tools/primer-blast/; accessed on 18 January 2023, [72]. The primers sequences used were as follows: Solyc02g084840, forward 5′-AACTCAGGCTGGACACACCA and reverse 5′-GAACGGTGAAGCATGCCACC; SlEF1A (housekeeping), forward 5′-GCTGCAGCTGTCACTGCTAA and reverse 5′-TACCCGGTGAACCATCTGGC.

All amplification plots were analyzed with the CFX Maestro software 1.1 (Bio-rad) to obtain Ct values. The relative expression levels of each gene were estimated by using the 2^−ΔΔCT^ method [71]. Relative expression of each gene was reported as the number of fold increase of the transcript level at each time point, compared to the control condition (W_WW). Three biological samples per treatment were analyzed with three technical replicates per sample.

### 4.8. Statistical Analyses

Each experiment was repeated thrice, and the mean values, standard deviations and standard error were calculated. Data were subjected to two-way ANOVA and Tukey’s HSD test for evaluating the differences among means at *p* ≤ 0.05. Mean values of treatments that were significantly different from each other were indicated by different letters.

## 5. Conclusions

*C. crispus* has been known for years for the production of carrageenans. However, its use as a biostimulant has not yet been investigated. This study represents the first demonstration of *C. crispus* extracts application as a method to improve tomato growth and drought resistance. Our finding showed that a protein hydrolysate-rich extract, by-product of the carrageenan extraction process from *C. crispus*, was effective at improving tomato growth. All measured tomato growth parameters were positively influenced by the *C. crispus* extract application. Moreover, the foliar application of *C. crispus* extract has been shown to be able to increase drought stress tolerance of tomato plants. This study also provides insights into the functional role of *C. crispus* extract in enhancing drought tolerance by changing ABA and proline levels, as well as the expression of protective genes, such as Solyc02g084840.

Finally, this work adds a piece to the current knowledge on the protein hydrolysate-induced biostimulating effect on plants. The label-free mass spectrometric characterization of proteins contained in *C. crispus* extracts tested in this study revealed a high abundance of pigment-protein complexes, such as phycoerythrins and phycocyanins, showing their possible use as crops biostimulants.

## Figures and Tables

**Table 1:** List of the top ten proteins identified by label free quantification (LFQ) intensity with their respective protein ID and peptides number.

Protein IDs	Description	Peptide Counts	LFQ Intensity
M5DDI2	R-phycoerythrin class I beta subunit	6	214,290,000
M5DDJ6	Ribulose bisphosphate carboxylase large chain	6	150,300,000
M5DDK1	Phycocyanin, alpha chain	5	50,310,000
M5DDI6	Allophycocyanin, beta chain	9	38,074,000
M5DDI5	Phycocyanin, beta chain	5	28,995,000
M5DDJ9	R-phycoerythrin class I alpha subunit	3	26,409,000
M5DBY2	ATP synthase subunit alpha	6	17,255,000
R7QSM0	Fructose-bisphosphate aldolase	4	17,039,000
R7QMR3	ATP synthase subunit alpha	7	11,272,000

## Data Availability

Data is contained within the article or Supplementary Materials.

## Supplementary Materials

The following supporting information can be downloaded at: https://www.mdpi.com/article/10.3390/plants12040845/s1, Table S1. Determination of the total protein concentration in each Chondrus crispus extract by the Bradford method; Table S2. List of protein groups identified by LC-MS/MS analysis in the extracts obtained by the hydrothermal treatment of *Chondrus crispus* with subcritical water at different temperatures (120–200 °C); Table S3. List of peptides identified by LC-MS/MS analysis in the extracts obtained by the hydrothermal treatment of *Chondrus crispus* with subcritical water at 160 °C.

## References

[1] Arora N.K. (2019). Impact of climate change on agriculture production and its sustainable solutions. Environ. Sustain..

[2] Placide R., Hirut G.B., Stephan N., Fekadu B. (2014). Assessment of drought stress tolerance in root and tuber crops. Afr. J. Plant Sci..

[3] Bhushan L.S., Pathma J. (2021). Impact of agro-chemicals on environment: A global perspective. Plant Cell Biotechnol. Mol. Biol..

[4] Caradonia F., Battaglia V., Righi L., Pascali G., La Torre A. (2018). Plant Biostimulant Regulatory Framework: Prospects in Europe and Current Situation at International Level. J. Plant Growth Regul..

[5] Rouphael Y., Colla G. (2020). Toward a sustainable agriculture through plant biostimulants: From experimental data to practical applications. Agronomy.

[6] Pacheco D., Cotas J., Rocha C.P., Araújo G.S., Figueirinha A., Gonçalves A.M., Bahcevandziev K., Pereira L. (2021). Seaweeds’ carbohydrate polymers as plant growth promoters. Carbohydr. Polym. Technol. Appl..

[7] Ali O., Ramsubhag A., Jayaraman J. (2021). Biostimulant Properties of Seaweed Extracts in Plants: Implications towards Sustainable Crop Production. Plants.

[8] Shukla P.S., Mantin E.G., Adil M., Bajpai S., Critchley A.T., Prithiviraj B. (2019). Ascophyllum nodosum-Based Biostimulants: Sustainable Applications in Agriculture for the Stimulation of Plant Growth, Stress Tolerance, and Disease Management. Front. Plant Sci..

[9] Okolie C.L., Mason B., Critchley A.T. (2018). Novel Proteins for Food, Pharmaceuticals and Agriculture.

[10] Deolu-Ajayi A.O., van der Meer I.M., van der Werf A., Karlova R. (2022). The power of seaweeds as plant biostimulants to boost crop production under abiotic stress. Plant Cell Environ..

[11] Carmody N., Goñi O., Łangowski Ł., O’Connell S. (2020). Extract Biostimulant Processing and Its Impact on Enhancing Heat Stress Tolerance During Tomato Fruit Set. Front. Plant Sci..

[12] Łangowski Ł., Goñi O., Marques F.S., Hamawaki O.T., da Silva C.O., Nogueira A.P.O., Teixeira M.A.J., Glasenapp J.S., Pereira M., O’Connell S. (2021). Ascophyllum nodosum Extract (SealicitTM) Boosts Soybean Yield Through Reduction of Pod Shattering-Related Seed Loss and Enhanced Seed Production. Front. Plant Sci..

[13] Shukla P.S., Borza T., Critchley A.T., Prithiviraj B. (2016). Carrageenans from Red Seaweeds as Promoters of Growth and Elicitors of Defense Response in Plants. Front. Mar. Sci..

[14] Abad L.V., Aurigue F.B., Relleve L.S., Montefalcon D.R.V., Lopez G.E. (2016). P, Characterization of low molecular weight fragments from gamma irradiated κ-carrageenan used as plant growth promoter. Radiat. Phys. Chem..

[15] Vera J., Castro J., Contreras R.A., González A., Moenne A. (2012). Oligo-carrageenans induce a long-term and broad-range pro-tection against pathogens in tobacco plants (var. Xanthi). Physiol. Mol. Plant Pathol..

[16] Trivedi K., Anand K.G.V., Kubavat D., Ghosh A. (2022). Role of *Kappaphycus alvarezii* seaweed extract and its active constituents, glycine betaine, choline chloride, and zeatin in the alleviation of drought stress at critical growth stages of maize crop. J. Appl. Phycol..

[17] Colla G., Nardi S., Cardarelli M., Ertani A., Lucini L., Canaguier R., Rouphael Y. (2015). Protein hydrolysates as biostimulants in horticulture. Sci. Hortic..

[18] Colla G., Hoagland L., Ruzzi M., Cardarelli M., Bonini P., Canaguier R., Rouphael Y. (2017). Biostimulant Action of Protein Hydrolysates: Unraveling Their Effects on Plant Physiology and Microbiome. Front. Plant Sci..

[19] Rouphael Y., Carillo P., Cristofano F., Cardarelli M., Colla G. (2021). Effects of vegetal-versus animal-derived protein hydrolysate on sweet basil morpho-physiological and metabolic traits. Sci. Hortic..

[20] Ponthier E., Domínguez H., Torres M.D. (2020). The microwave assisted extraction sway on the features of antioxidant compounds and gelling biopolymers from *Mastocarpus stellatus*. Algal Res..

[21] Álvarez-Viñas M., González-Ballesteros N., Torres M.D., López-Hortas L., Vanini C., Domingo G., Rodríguez-Argüelles M.C., Domínguez H. (2022). Efficient extraction of carrageenans from *Chondrus crispus* for the green synthesis of gold nanoparticles and formulation of printable hydrogels. Int. J. Biol. Macromol..

[22] Solankey S.S., Singh R.K., Baranwal D.K., Singh D.K. (2015). Genetic Expression of Tomato for Heat and Drought Stress Tolerance: An Overview. Int. J. Veg. Sci..

[23] Zilinskas B.A., Greenwald L.S. (1986). Phycobilisome structure and function. Photosynth. Res..

[24] Mariani L., Ferrante A. (2017). Agronomic management for enhancing plant tolerance to abiotic stresses—Drought, salinity, hypoxia, and lodging. Horticulturae.

[25] Goñi O., Quille P., O’Connell S. (2018). Ascophyllum nodosum extract biostimulants and their role in enhancing tolerance to drought stress in tomato plants. Plant Physiol. Biochem..

[26] Jacomassi L.M., Viveiros J.D.O., Oliveira M.P., Momesso L., de Siqueira G.F., Crusciol C.A.C. (2022). A Seaweed Extract-Based Biostimulant Mitigates Drought Stress in Sugarcane. Front. Plant Sci..

[27] El Boukhari M.E.M., Barakate M., Bouhia Y., Lyamlouli K. (2020). Trends in Seaweed Extract Based Biostimulants: Manufacturing Process and Beneficial Effect on Soil-Plant Systems. Plants.

[28] Rady M.M., Taha R.S., Mahdi A.H. (2016). Proline enhances growth, productivity and anatomy of two varieties of *Lupinus termis* L. grown under salt stress. S. Afr. J. Bot..

[29] Roy D., Basu N., Bhunia A., Banerjee S.K. (1993). Counteraction of exogenous L-proline with NaCl in salt-sensitive cultivar of rice. Biol. Plant..

[30] Hayat S., Hayat Q., Alyemeni M.N., Wani A.S., Pichtel J., Ahmad A. (2012). Role of proline under changing environments: A review. Plant Signal. Behav..

[31] Ma Y., Qin F. (2014). ABA regulation of plant responses to drought and salt stresses. Abscisic Acid: Metabolism, Transport and Signaling.

[32] Iuchi S., Kobayashi M., Taji T., Naramoto M., Seki M., Kato T., Tabata S., Kakubari Y., Yamaguchi-Shinozaki K., Shinozaki K. (2001). Regulation of drought tolerance by gene manipulation of 9-cis-epoxycarotenoid dioxygenase, a key enzyme in abscisic acid biosynthesis in Arabidopsis. Plant J..

[33] Sharma S., Chen C., Khatri K., Rathore M.S., Pandey S.P. (2019). *Gracilaria dura* extract confers drought tolerance in wheat by modulating abscisic acid homeostasis. Plant Physiol. Biochem..

[34] Bharath P., Gahir S., Raghavendra A.S. (2021). Abscisic Acid-Induced Stomatal Closure: An Important Component of Plant Defense Against Abiotic and Biotic Stress. Front. Plant Sci..

[35] Raghavendra A.S., Gonugunta V.K., Christmann A., Grill E. (2010). ABA perception and signalling. Trends Plant Sci..

[36] Santaniello A., Scartazza A., Gresta F., Loreti E., Biasone A., Di Tommaso D., Piaggesi A., Perata P. (2017). *Ascophyllum nodosum* Seaweed Extract Alleviates Drought Stress in Arabidopsis by Affecting Photosynthetic Performance and Related Gene Expression. Front. Plant Sci..

[37] Rasul F., Gupta S., Olas J.J., Gechev T., Sujeeth N., Mueller-Roeber B. (2021). Priming with a Seaweed Extract Strongly Improves Drought Tolerance in Arabidopsis. Int. J. Mol. Sci..

[38] Liu H., Zhang Y.-H., Yin H., Wang W.-X., Zhao X.-M., Du Y.-G. (2013). Alginate oligosaccharides enhanced *Triticum aestivum* L. tolerance to drought stress. Plant Physiol. Biochem..

[39] Wally O.S.D., Critchley A.T., Hiltz D., Craigie J.S., Han X., Zaharia L.I., Abrams S.R., Prithiviraj B. (2012). Regulation of Phytohormone Biosynthesis and Accumulation in Arabidopsis Following Treatment with Commercial Extract from the Marine Macroalga Ascophyllum nodosum. J. Plant Growth Regul..

[40] Petrozza A., Santaniello A., Summerer S., Di Tommaso G., Di Tommaso D., Paparelli E., Piaggesi A., Perata P., Cellini F. (2014). Physiological responses to Megafol^®^ treatments in tomato plants under drought stress: A phenomic and molecular approach. Sci. Hortic..

[41] Sun Z., Li S., Chen W., Zhang J., Zhang L., Sun W., Wang Z. (2021). Plant Dehydrins: Expression, Regulatory Networks, and Protective Roles in Plants Challenged by Abiotic Stress. Int. J. Mol. Sci..

[42] Wang Y., Xu H., Zhu H., Tao Y., Zhang G., Zhang L., Zhang C., Zhang Z., Ma Z. (2014). Classification and expression diversification of wheat dehydrin genes. Plant Sci..

[43] Hoque T.S., Uraji M., Tuya A., Nakamura Y., Murata Y. (2012). Methylglyoxal inhibits seed germination and root elongation and up-regulates transcription of stress-responsive genes in ABA-dependent pathway in Arabidopsis. Plant Biol..

[44] Graether S.P., Boddington K.F. (2014). Disorder and function: A review of the dehydrin protein family. Front. Plant Sci..

[45] Eriksson S.K., Harryson P., Lüttge U., Beck E., Bartels D. (2011). Plant Desiccation Tolerance.

[46] Yoshida T., Fujita Y., Sayama H., Kidokoro S., Maruyama K., Mizoi J., Shinozaki K., Yamaguchi-Shinozaki K. (2010). AREB1, AREB2, and ABF3 are master transcription factors that cooperatively regulate ABRE-dependent ABA signaling involved in drought stress tolerance and require ABA for full activation. Plant J..

[47] Khan W., Rayirath U.P., Subramanian S., Jithesh M.N., Rayorath P., Hodges D.M., Critchley A.T., Craigie J.S., Norrie J., Prithiviraj B. (2009). Seaweed Extracts as Biostimulants of Plant Growth and Development. J. Plant Growth Regul..

[48] Sharma H.S.S., Fleming C., Selby C., Rao J.R., Martin T. (2014). Plant biostimulants: A review on the processing of macroalgae and use of extracts for crop management to reduce abiotic and biotic stresses. J. Appl. Phycol..

[49] Craigie J.S. (2011). Seaweed extract stimuli in plant science and agriculture. J. Appl. Phycol..

[50] Michalak I., Chojnacka K. (2014). Algal extracts: Technology and advances. Eng. Life Sci..

[51] Morales-Payan J.P., Stall W. (2003). Papaya (*Carica papaya*) response to foliar treatments with organic complexes of peptides and amino acids. Proc. Fla. State Hortic. Soc..

[52] Parrado J., Escudero-Gilete M.L., Friaza V., García-Martínez A., González-Miret M.L., Bautista J.D., Heredia F.J. (2007). Enzymatic vegetable extract with bio- active components: Influence of fertiliser on the colour and anthocyanins of red grapes. J. Sci. Food Agri..

[53] Kowalczyk K., Zielony T., Marek G., Dąbrowski Z.T. (2008). Effect of Aminoplant and Asahi on yield and quality of lettuce grown on rockwool. Biostimulators in Modern Agriculture: Vegetable Crops.

[54] Ertani A., Schiavon M., Muscolo A., Nardi S. (2013). Alfalfa plant-derived biostimulant stimulate short-term growth of salt stressed *Zea mays* L. plants. Plant Soil.

[55] Colla G., Rouphael Y., Canaguier R., Svecova E., Cardarelli M. (2014). Biostimulant action of a plant-derived protein hydrolysate produced through enzymatic hydrolysis. Front. Plant Sci..

[56] Rouphael Y., Colla G., Giordano M., El-Nakhel C., Kyriacou M.C., De Pascale S. (2017). Foliar applications of a legume-derived protein hydrolysate elicit dose-dependent increases of growth, leaf mineral composition, yield and fruit quality in two greenhouse tomato cultivars. Sci. Hortic..

[57] Barrada A., Delisle-Houde M., Nguyen T.T., Tweddell R.J., Dorais M. (2022). Drench Application of Soy Protein Hydrolysates Increases Tomato Plant Fitness, Fruit Yield, and Resistance to a Hemibiotrophic Pathogen. Agronomy.

[58] Ito Y., Nakanomyo I., Motose H., Iwamoto K., Sawa S., Dohmae N., Fukuda H. (2006). Dodeca-CLE Peptides as Suppressors of Plant Stem Cell Differentiation. Science.

[59] Kondo T., Sawa S., Kinoshita A., Mizuno S., Kakimoto T., Fukuda H., Sakagami Y. (2006). A Plant Peptide Encoded by CLV3 Identified by in Situ MALDI-TOF MS *Anal*. Science.

[60] Vani L., Ciavatta C. (2007). Attività Biostimolante degli Idrolizzati Proteici. L’informatore Agrar..

[61] Matsumiya Y., Kubo M. (2011). Soybean peptide: Novel plant growth promoting peptide from soybean. Soybean and Nutrition.

[62] Varia J., Kamaleson C., Lerer L. (2022). Biostimulation with phycocyanin-rich Spirulina extract in hydroponic vertical farming. Sci. Hortic..

[63] Korkmaz A., Ahmad P., Prasad M.N.V. (2012). Abiotic Stress Responses in Plants: Metabolism, Productivity and Sustainability.

[64] Hotta Y., Tanaka T., Takaoka H., Takeuchi Y., Konnai M. (1997). Promotive effects of 5-aminolevulinic acid on the yield of several crops. Plant Growth Regul..

[65] Watanabe K., Nishihara E., Watanabe S., Tanaka T., Takahashi K., Takeuchi Y. (2006). Enhancement of Growth and Fruit Maturity in 2-year-old Grapevines cv. Delaware by 5-Aminolevulinic Acid. Plant Growth Regul..

[66] Kruger N.J., Walker J.M. (2009). The Bradford Method for Protein Quantitation. The Protein Protocols Handbook.

[67] Vannini C., Marsoni M., Scoccianti V., Ceccarini C., Domingo G., Bracale M., Crinelli R. (2019). Proteasome-mediated remodeling of the proteome and phospho-proteome during kiwifruit pollen germination. J. Proteom..

[68] Mancini I., Domingo G., Bracale M., Loreto F., Pollastri S. (2022). Isoprene Emission Influences the Proteomic Profile of Arabidopsis Plants under Well-Watered and Drought-Stress Conditions. Int. J. Mol. Sci..

[69] Aranda P.S., LaJoie D.M., Jorcyk C.L. (2012). Bleach gel: A simple agarose gel for analyzing RNA quality. Electrophoresis.

[70] Ding Y., Fromm M., Avramova Z. (2012). Multiple exposures to drought ‘train’ transcriptional responses in Arabidopsis. Nat. Commun..

[71] Livak K.J., Schmittgen T.D. (2001). Analysis of relative gene expression data using real-time quantitative PCR and the 2(-Delta C(T)) Method. Methods.

[72] Ye J., Coulouris G., Zaretskaya I., Cutcutache I., Rozen S., Madden T.L. (2012). Primer-BLAST: A tool to design target-specific primers for polymerase chain reaction. BMC Bioinform..

